# Hepatitis A Outbreak in Skilled Nursing Facility, Los Angeles County, California, USA, 2025

**DOI:** 10.3201/eid3208.260202

**Published:** 2026-08

**Authors:** Mona T. Yazdi, Alicia H. Chang, Angela Wong, Christopher Asuncion, Bonnie Dao, Prabhu P. Gounder

**Affiliations:** Los Angeles County Department of Public Health, Los Angeles, California, USA

**Keywords:** hepatitis A, viruses, nursing homes, vaccine, post-exposure prophylaxis, Immune globulin, California, United States

## Abstract

We investigated an outbreak of 5 hepatitis A cases among skilled nursing facility residents in Los Angeles County, California, USA, including 3 who were vaccinated after exposure. Our results suggest that persons in congregate living facilities should receive both the vaccine and immune globulin after hepatitis A exposure to mitigate further transmission.

Hepatitis A (HepA) is a communicable disease of the liver caused by hepatitis A virus (HAV), which can easily spread between persons in congregate living facilities. A person with acute hepatitis A is considered infectious 14 days before and up to 7 days after jaundice onset, or 14 days after symptom onset if there is no jaundice. The HepA vaccine is recommended for postexposure prophylaxis (PEP) within 14 days of exposure, during the infectious period, if no prior immunity is documented. Immune globulin (IG) can be considered for PEP in persons with special risk factors for either HAV infection or severe disease from HAV infection (*1*).

Since January 2024, Los Angeles County (LAC), California, USA, experienced an increase in community transmission of HepA. Initially, HepA cases were predominantly among persons experiencing homelessness, who use drugs, or both. By 2025, however, most cases in LAC were in persons without risk factors for HAV infection. We describe an outbreak of HepA among skilled nursing facility (SNF) residents and the public health effort to mitigate spread.

In January 2025, the LAC Department of Public Health (DPH) was notified of a HepA case in an LAC SNF with 5 separate units and 285 residents, all >40 years of age. Medical providers and laboratories are mandated to report HepA cases to LAC DPH. LAC DPH investigators reviewed medical records to determine if reports met the Council of State and Territorial Epidemiologists’ surveillance definition of a confirmed HepA case (*2*). When possible, LAC DPH also obtained blood samples from reported cases for HepA PCR confirmatory testing.

We identified the first HepA case (case A) in a closed 38-bed unit intended for patients with cognitive or behavioral health needs (unit 1) (Figure 1). After the first case was identified, LAC DPH staff administered the HepA vaccine to all 34 residents without documented immunity in unit 1 (Figure 2). However, 2 additional cases of HepA (cases B and C) were identified in unit 1 after HepA vaccination and were reported to DPH (Figure 1). We measured total HAV antibodies in the serum of 31 residents (1 resident did not provide a sample) who had not contracted HAV but received the HepA vaccine. Of those residents, 68% (n = 21) had protective HAV antibody levels (>10 mIU/mL). We administered IG to 10 residents who did not develop protective antibody levels and to the 1 resident not tested.

**Figure 1:**
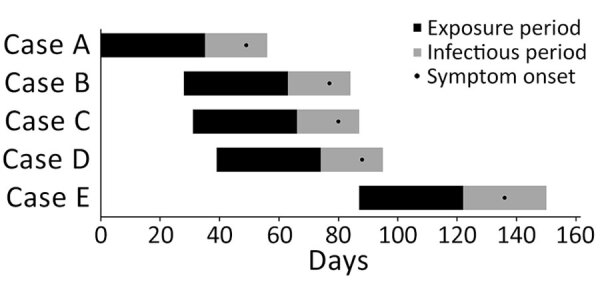
Epidemic curve of hepatitis A cases associated with a skilled nursing facility outbreak in Los Angeles County, California, USA, 2025 (N = 5). We defined the exposure period as 50 days before symptom onset and until onset of infectious period; we defined the infectious period as 14 days before symptom onset to 7 days after jaundice onset (or 14 days after symptom onset if no jaundice).

**Figure 2:**
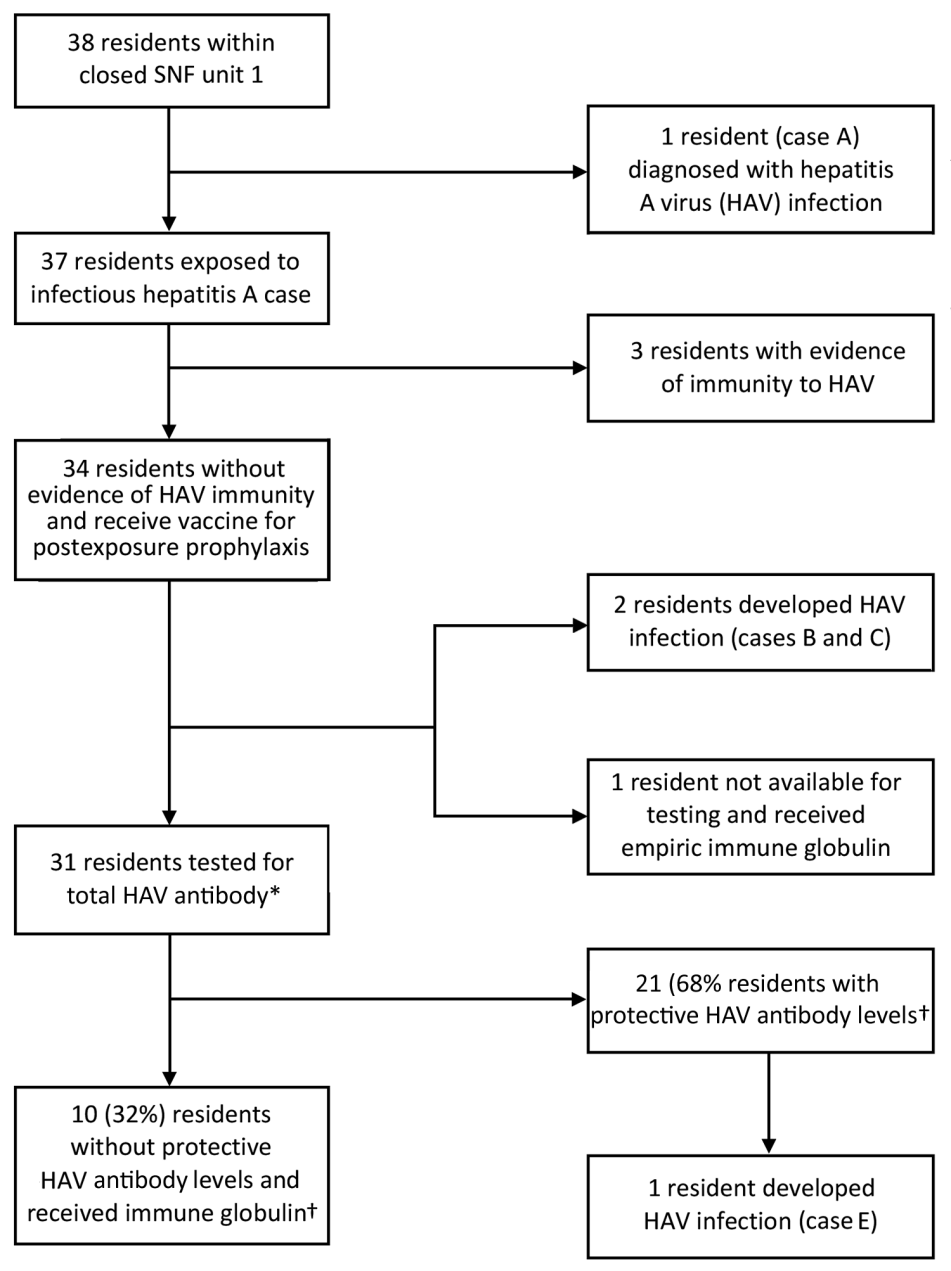
Outcomes of residents in skilled nursing facility closed unit 1 who were exposed to a resident with infectious HAV during an outbreak in a SNF, Los Angeles County, California, USA, 2025. Asterisk (*) indicates testing occurred 18 days after HAV vaccine postexposure prophylaxis. Dagger (†) indicates protective HAV total antibody levels that we defined as titer >10 mIU/mL. HAV, hepatitis A virus; SNF, skilled nursing facility.

We initially missed the next HepA case (case D) because the resident was in unit 1 and was transferred to unit 2 before the LAC DPH investigation (Figure 1). After case D was reported, LAC DPH staff administered the HepA vaccine and IG for PEP to the remaining 5 residents in unit 2. Subsequently, LAC DPH staff offered the HepA vaccine to residents in the 3 unaffected units and to all facility staff. Overall, 235 (82%) SNF residents and 10 (4%) facility staff members received the HepA vaccine. In addition, 16 residents from units 1 and 2 received IG. 

An additional case (case E) was reported to the LAC DPH 78 days after the initial case (Figure 1). Case E was a resident of unit 1, had received the HepA vaccine for PEP, and was seropositive after 1 dose. The exact infection onset was unknown because the resident did not have symptoms or clinical findings associated with acute hepatitis but had detectable HAV IgM and a positive HAV PCR result. All patients with HepA recovered, and no HAV infections were identified among SNF staff.

We investigated a HepA outbreak among residents of a SNF, an uncommon setting for HAV infection (*3*). Cases B, C, and E received 1 dose of the HepA vaccine for PEP, and case E had detectable serum HAV antibodies. Those cases might have already had established infections before receiving PEP and might not represent prophylaxis failure. However, our finding of incomplete early (<14 days) immunity after a single dose of the HepA vaccine for PEP is consistent with prior reports (*4*). Those results support offering concurrent IG to all exposed persons in congregate settings who have an indicated condition associated with reduced early immune response to the HepA vaccine alone (*1*). In addition, anyone exposed to HepA who did not develop the disease should get a second dose of the HepA vaccine >6 months after the first, to ensure lasting immunity against HAV. 

Our findings should also prompt increased surveillance for HepA in congregate living facility residents during periods of elevated community transmission. Under those circumstances, providers could consider routinely offering the HepA vaccine to all residents and staff in congregate living facilities to reduce the risk infection and transmission, particularly because HAV seropositivity among adults has declined since 1996 (*5*,*6*).
